# Effect of Paroxetine on the Neuropathic Pain: A Molecular Study

**DOI:** 10.29252/ibj.24.5.301

**Published:** 2020-05-02

**Authors:** Malek Zarei, Masoumeh Sabetkasaei, Taraneh Moini-Zanjani

**Affiliations:** Department of Pharmacology, Faculty of Medicine, Shahid Beheshti University of Medical Sciences, Tehran, Iran

**Keywords:** Brain-derived neurotrophic factor, Gamma-aminobutyric acid, Paroxetine

## Abstract

**Background:**

Neuropathic pain, due to peripheral nerve damage, has influenced millions of people living all over the world. It has been shown that paroxetine can relieve neuropathic pain. Recently, the role of certain proteins like *BDNF*, *GABAA* receptor, and *KCC2* transporter in the occurrence of neuropathic pain has been documented. In the current study, the expression of these proteins affected by paroxetine was evaluated.

**Methods:**

Male Wistar rats were allocated into two main groups of pre- and post-injury. Rats in each main group received paroxetine before nerve injury and at day seven after nerve damage till day 14, respectively. The lumbar spinal cord of animals was extracted to assess the expression of target genes and proteins.

**Results:**

In the preventive study, paroxetine decreased *BDNF* and increased *KCC2* and *GABAA* gene and protein expression, while in the post-injury paradigm, it decreased *BDNF* and increased *KCC2* genes and protein expression. In this regard, an increase in the protein expression of *GABAA* was observed.

**Conclusion:**

It seems that paroxetine with a change in the expression of three significant proteins involved in neuropathic pain could attenuate this type of chronic pain.

## INTRODUCTION

Neuropathic pain occurs by damage to the somatosensory system, including peripheral and central neurons, through some diseases like diabetes mellitus, trauma, and cancer^[^^1 ▶^^]^. Although the aspects about the management of neuropathic pain have been changed, there are limited data on the pathogenesis of this pain^[^^2 ▶^^,^^3 ▶^^]^. In spite of the recent progress in neurosciences and pharmaceutical technology, no effective drug, with a clear mechanism, has been developed to manage the neuropathic pain^[^^4 ▶^^]^.

Among different mechanisms involved in the neuropathic pain, the *GABAergic *system is the key player. *GABA* receptors located in pre- and post-synaptic terminals of primary afferent neurons are also found in the dorsal horn laminae I-IV^[^^5 ▶^^]^. Dorsal horn *GABAergic* interneurons play an important role in decreasing pain^[^^6 ▶^^,^^7 ▶^^]^. So far, the relationship between the *GABAergic* system and neuropathic pain has not been understood well^[^^8 ▶^^]^. It has been shown that *GABAergic* neurons transplanted to subarachnoid space attenuate hyperalgesia produced by nerve damage in rat^[^^9 ▶^^]^. Moreover, muscimol, as a *GABAA* receptor agonist decreases hyperalgesia caused by peripheral neurons injury^[^^10 ▶^^]^. The normal function of the *GABAergic* system is extremely dependent on cation-chloride cotransporters. The influx and efflux of chloride into and out of neurons are facilitated mostly by NKCC1 and *KCC2*, respectively^[^^11 ▶^^,^^12 ▶^^]^. Both NKCC1 and *KCC2* expressed in spinal cord regulate intracellular chloride homeostasis. Various studies have indicated that the altered expression of these transporters can change neuropathic pain behavior^[^^6 ▶^^,^^7 ▶^^,^^13 ▶^^,^^14 ▶^^]^. On the other hand, the elevated concentration of intracellular chloride diminishes the inhibitory effect of *GABA* receptors^[^^8 ▶^^]^.

New data show that glial cells in spinal cord cause hypersensitivity and continuation of pain in CNS^[^^15 ▶^^]^. Nerve injury not only increases specific microglia markers expression *(Iba1)* but also releases some painful mediators such as BDNF, prostaglandin E2, nitric oxide, and tumor necrosis factor-α. These factors produce hypersensitivity in CNS through rising the excitability and decreasing the inhibition of DRG neurons^[^^16 ▶^^]^. Most of these mediators have a significant role in the production of chronic pain^[^^17 ▶^^]^.

Damage to nerve activates p38MAPK pathway in DRG and microglial cells^[^^18 ▶^^]^. A recent study has suggested that the activation of the p38MAPK pathway by ATP and purinergic P2X4 receptor in microglia results in the production and release of *BDNF*^[^^19 ▶^^]^. Investigations have also displayed that *BDNF* has a prominent function in neuropathic pain. Thermal hyperalgesia and mechanical allodynia are induced by intrathecal administration of *BDNF*^[^^20 ▶^^]^. Other studies have revealed that nerve injury and peripheral inflammation change gene expression and production of *BDNF*^[^^21 ▶^^,^^22 ▶^^]^. BDNF has ability to change the function of *GABAA* receptor^[^^23 ▶^^]^ and also alters *KCC2* gene expression, thereby effluxing chloride from the neuron; these changes finally result in chloride efflux through *GABAA* and accordingly, depolarization of neurons^[^^24 ▶^^]^. Based on evidence, alteration in *KCC2* expression gives rise to a change in *GABAA* behavior from inhibitory to excitatory in some subtypes of injured nerves^[^^6 ▶^^]^. The *GABAA* receptor subtypes α_2 _and γ_2 _is expressed mostly in the spinal dorsal horn. Although γ_2 _subunit gene expression decreases after nerve injury, α_2_ subtype expression has no significant changes^[^^25 ▶^^]^.

Considering P2X4 receptors role in the release of important mediators involved in neuropathic pain, inhibition of this receptor can be helpful to understand the mechanism of neuropathic pain. Up to now, no selective inhibitor of the P2X4 receptor has been presented^[^^26 ▶^^]^. Antidepressant drugs, specifically tricyclic antidepressants have been widely used to manage neuropathic pain. It has been demonstrated that some antidepressants and antiseizure drugs are applied to treat neuropathic pain and inhibit the P2X4 receptor^[^^27 ▶^^]^. Among the antidepressants, paroxetine has a significant inhibitory effect on P2X4 receptors^[^^28 ▶^^,^^29 ▶^^]^. In the current study, we aimed to find out any possible changes in the expression of some proteins involved in the neuropathic pain *(Iba1*, *BDNF*,* KCC2*, and* GABAA/γ*_2_*)* affected by paroxetine. 

## MATERIALS AND METHODS

**Animals**

Rats (male Wistar, 150-200 g) used in the study were housed in an environment with controlled temperature (23 ± 2 °C). Food and water were available to animals without any limitation. At least one week before surgery, all the rats were permitted to be adapted to the housing facilities. 

**Surgery and drug preparation**

The left sciatic nerve close to trifurcation was tied loosely (4 ligatures) by chromic gut suture, and thus, a model of neuropathic pain so-called CCI was created. Except for the sham group, the left sciatic nerve was tied in both drug and control groups. After ligation, the wound was closed. All surgical procedures were under sterile condition. Ketamine (60 mg/kg) and xylazine (10 mg/kg) were administered for the induction of aanesthesia. Paroxetine hydrochloride (Sigma, USA) was dissolved in DMSO 5%. 

**Drug administration**

Animals were placed to pre- and post-injury groups. In each group, the rats were divided into CCI vehicle-treated (control), sham, and CCI paroxetine-treated groups. CCI- and sham-operated animals received the vehicle. Paroxetine was administered (i.p.) to the drug-treated group before and after surgery. In the pre-emptive paradigm, 10 mg/kg of paroxetine was injected to rats one hour prior to surgery and then daily after surgery until day 14. Animals in the post-injury group received the drug the same dose as the preventive group at day seven post-injury and then daily until day 14.

**Tissue collection for RT-PCR and Western blot analysis**

After euthanizing by CO_2_ asphyxiation, rats were decapitated immediately on day 14 post surgery. The spinal cord displaced by the normal saline from the vertebral column was frozen in dry ice. For evaluating gene and protein, the lumbar spinal cord segment was isolated from the intact frozen cord.

**Gene expression study**

 Isothiocyanate-phenol-chloroform protocol was used for the isolation of total RNA, using RNX+ reagent (Cinaclon, Iran), according to the instructions provided by the manufacturer. Based on the manufacturer's protocol, 2 μg of total RNA, Oligo(dT) primer (Fermentas, USA), and M-MuLV reverse transcriptase (Fermentas) was used for the synthesis of cDNA. As shown in Table 1, designing of primer sequences (CinnaGen, Iran) was performed as per sequences in the GenBank. The PCR was carried out using the synthesized cDNA, the specific primers, and Taq DNA Polymerase MasterMix. In the beginning, the PCR was run for 10 min (95 °C), then continued by amplification cycles (25 or 26), each including 1-min denaturation (95 °C), annealining(45 s, 59 °C), and extension (45 s, 72 °C) steps. PCR products were separated on agarose gel (1.5%; Roche, Germany), stained with SYBR Green staining and visualized under a UV light. The PCR bands intensities, as an indicator of the gene expression levels, were measured by the Laboratory Works software version 4 (UVP, UK). 

**Table 1:** Primer sequences for the PCR amplification of genes of interest

**Primer length (bp)**	**Sequence 5** ** to 3**	**Primer ** **name**	**Target ** **gene**
21 23	5 ACAAGCACTTCCTCGATGATC 3 5 GCAACTCAGAAATAGCTTTCTTG 3	F-iba1 R-iba1	*Iba1*
			
20 21	5 GCTGCGCCCATGAAAGAAGC 3 5 GAACCCTCATCGACATGTTTG 3	F-bdnf R-bdnf	*BDNF*
			
22 20	5 AGGAGGAGATGGACACCAGCCC 3 5 GCGTAGATGGCCAGGCCAGG 3	F-kcc2 R-kcc2	*KCC2*
22 21	5 AAGATTATGCTTCTAATAAAAC 3 5 CACCATATTGCTATTCAATCG 3	F-gaba-a R-gaba-a	*GABAA/2*
			
23 23	5 GTTACCAGGGCTGCCTTCTCTTG 3 5 GTGGTGCAGGATGCATTGCTGAC 3	F-gapdh R-gapdh	*GAPDH*

**Western blot analysis**

For protein expression assay, Western blotting was used. Tissue samples were lysed in RIPA buffer (150 mM of NaCl, 1% NP-40, 50 mM of Tris pH 8.0, 1% SDS, 0.5% sodium deoxycholate, and 1 mM of EDTA and protease inhibitor cocktail) and centrifuged at 20,000 ×g at 4 ºC for 20 min. After adding the SDS sample buffer to the aliquots of tissue extracts, the samples were placed in a water bath at 100 ºC for 5 min. Proteins were separated on 10% SDS-PAGE and then transferred to the blot. Blot membranes were incubated with 1:500 dilution of specific primary polyclonal rabbit antibodies against *BDNF*,* KCC2*, *GABAA/γ*_2_, and *Iba1* and 1:1000 dilution of *GAPDH* (all from Abcam, USA) in TBS-T for 18 h. Blots were then incubated separately with secondary anti-rabbit (1:500 dilution; Abcam) in TBS-T for 90 min. *KCC2*, *GABAA/γ*_2_, *BDNF*, and *Iba1* immune-reactive proteins were detected using achemiluminescence kit (Enhanced Chemiluminescence, Amersham Biosciences, UK). The signal intensity of the detected bands was measured by an image analysis system (Image j, version 1.46r).

**Statistical analysis**

Statistical analyses were performed by SPSS 18 software using ANOVA, followed by Tukey’s post hoc test. Statistical significance was indicated by *p* < 0.05.

**Ethical statement**

The above-mentioned sampling and treatment protocols were approved by the Research Ethics Committee of Shahid Beheshti University of Medical Sciences, Tehran, Iran (ethical code: SBMU.REC1393. 327). 

## RESULTS

**Effect of prophylactic administration of paroxetine on the gene expression**

**Fig. 1:**
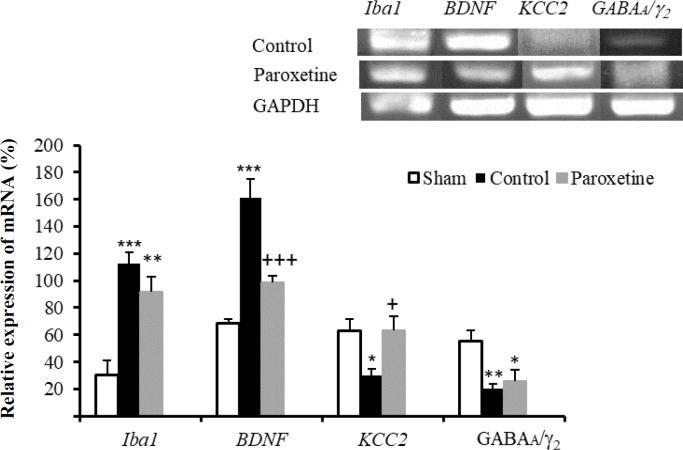
Effect of pre-injury administration of paroxetine on the gene expression of *Iba1*, *BDNF*,* KCC2*, and* GABAA/* _2_. *GAPDH* was used as a loading control. Relative expression of mRNA: band density of genes/*GAPDH* band density. Data are expressed as means ± SEM. ^*^p < 0.05, ^**^p < 0.01, and ^***^p < 0.001 indicate a statistically significant difference compared to the sham group, and ^+^*p* < 0.05 and ^+++^*p* < 0.001 show significant difference compared to the control group

Paroxetine and control groups showed a significant difference in the iba1 expression (*p *< 0.01 and *p *< 0.001, respectively) relative to the sham group Fig. 1). Compared to the control group, paroxetine decreased the expression of *BDNF* (*p* < 0.001). In comparison to the sham and control groups, the expression of *KCC2* decreased and increased significantly in the control (*p* < 0.05) and paroxetine (*p* < 0.05) groups. However, paroxetine decreased the gene expression of *GABAA/*_2_ (*p* < 0.05) compared to the sham group. 

**Effect of paroxetine on gene expression after nerve injury**

**Fig. 2:**
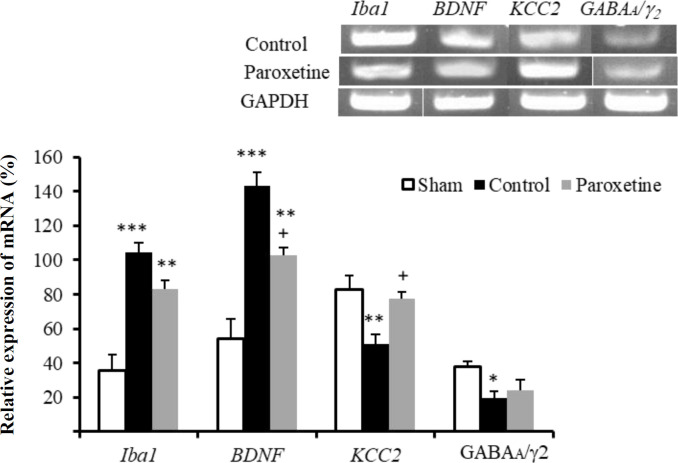
Effect of paroxetine on the gene expression of *Iba1*,* BDNF*,* KCC2*, and* GABAA/* _2_ administered after nerve injury. *GAPDH* was used as a loading control. Relative expression of mRNA: band density of genes/*GAPDH* band density. Data are expressed as means ± SEM. ^*^p < 0.05, ^**^p < 0.01, and ^***^p < 0.001 indicate a statistically significant difference when compared to the sham group, and ^+^*p* < 0.05 shows significant difference compared to the control group

As depicted in Figure 2, a significant rise was seen in the expression of Iba1in the control (*p* < 0.001) and paroxetine (*p* < 0.01) groups compared to the sham group. The paroxetine-treated group showed a significant decline in the expression of *BDNF* compared to the control (*p* < 0.05) and sham (*p* < 0.01) groups. *KCC2* gene expression showed a significant reduction in the control group in comparison to the sham group *p* < 0.01). Compared to the control, the paroxetine group showed a significant rise (^+^*p* < 0.05) in *KCC2* gene expression. Moreover, no significant decrease was observed in the expression of *GABAA/*  in the drug-treated group relative to other groups. 

**Effect of prophylactic injection of paroxetine target proteins**

**Fig. 3:**
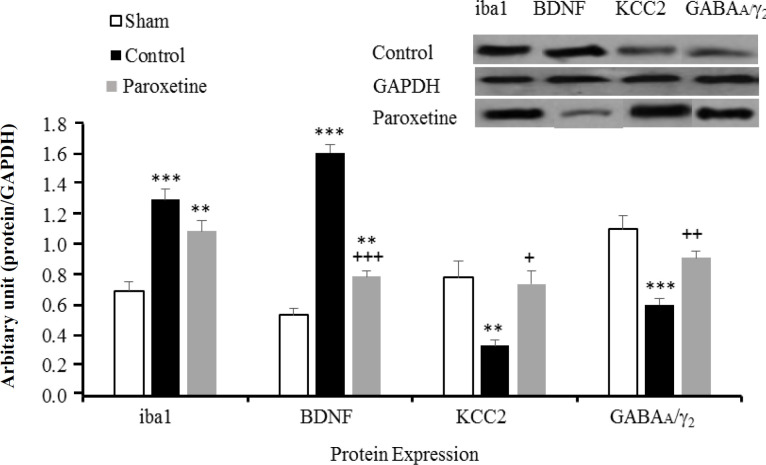
Effect of prophylactic injection of paroxetine on the protein expression levels of Iba1, BDNF, KCC2, and GABAA*/* _2_. GAPDH was used as a loading control. Arbitrary unit: proteins optical density/*GAPDH* optical density. Data are expressed as means ± SEM. ^**^p < 0.01 and ^***^p < 0.001 indicate a statistically significant difference when compared to the sham group, and ^+^*p* < 0.05, ^++^*p* < 0.001, and ^+++^*p* < 0.001 show significant difference compared to the control group

As it is clear from Figure 3, compared to the sham group, the expression level of the microglia marker *(*Iba1*)* rose in the control (*p* < 0.001) and paroxetine (*p* < 0.01) groups. Paroxetine could decrease the BDNF protein level significantly as compared to the sham (^**^p < 0.01) and the control (*p* < 0.001) groups. Compared to the control group, KCC2 showed a significant rise in the paroxetine group (*p* < 0.05). On the other hand, a significant difference in GABAA*/*_2_ levels (*p* < 0.001) was found between paroxetine and control group.

**Effect of paroxetine on the expression of target proteins after nerve injury**

**Fig. 4:**
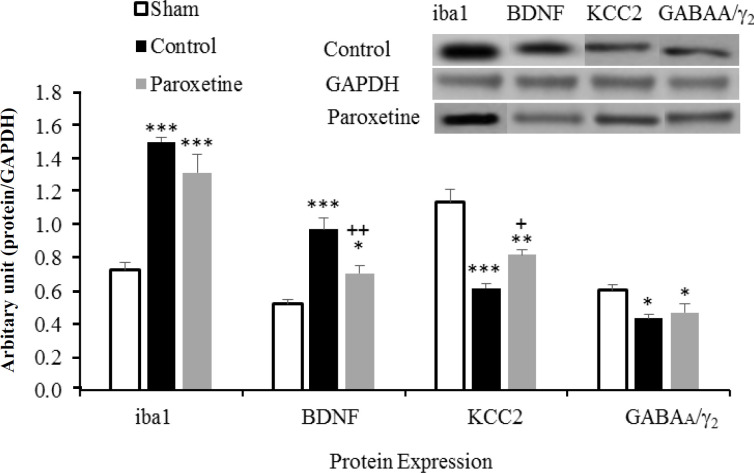
Effect of paroxetine on of Iba1, BDNF, KCC2, and GABAA*/* _2_ protein levels administered after nerve injury. *GAPDH* was used as a loading control. Arbitrary unit: proteins optical density/GAPDH optical density. Data are expressed as means ± SEM. ^*^p < 0.05, ^**^p < 0.01, and ^***^p < 0.001 indicate a statistically significant difference when compared to the sham group, and^ +^*p* < 0.05, ^++^*p* < 0.01 show significant difference compared to the control group

There was a significant rise in Iba1 protein level in the control and paroxetine groups as compared to the sham group (*p* < 0.001; Fig. 4). On the other hand, compared to the control group, the expression of BDNF protein decreased significantly in the paroxetine-treated group (*p* < 0.01). Paroxetine increased the KCC2 expression compared to the control group (*p* < 0.05). GABAA*/*_2_ protein levels showed no change in the drug-treated group in comparison with the control group.

## DISCUSSION

In the present study, the effect of paroxetine on the expression of certain important mediators involved in neuropathic pain was investigated. Our data indicated an altered expression of *BDNF* and *KCC2* upon administration of paroxetine. This was also the case for *GABAA/*_2_ proteins when the drug was injected before nerve damage. Microglial cells remain in resting state under physiological conditions and have appendages morphologically. Nerve injuries and lesions activate microglial cells and change their morphology with losing the appendages as well as swelling. After nerve injury, the gene expression level of P2X4 receptor and the Iba1 protein levels increased in microglial cells^[^^30 ▶^^,^^31 ▶^^]^. It has been shown that *Iba1* gene is specifically expressed in microglial cells in the CNS^[^^32 ▶^^]^ but not in other cells (neurons, astrocytes, and oligodendrocytes)^[^^33 ▶^^]^. Studies have evidenced the significant role of Iba1 in the migration and phagocytic activity of microglial cells^[^^34 ▶^^]^. Moreover, the expression of this protein and mechanical allodynia elevates after SNL in the rat^[^^35 ▶^^]^. As noted previously, paroxetine has the most effect on the inhibition of the P2X4 receptor among various antidepressants and antiepileptic drugs used in neuropathic pain. A previous study has indicated that the expression of this receptor enhances in microglia after the nerve injury, and the neuropathic pain symptoms are also observed more frequently afterward. However, there is no change in the expression of this receptor in nerve cells or astrocytes^[^^36 ▶^^]^. Another report has suggested that the P2X4 ionotropic receptor is expressed only in microglia and its expression increases after neuropathy^[^^37 ▶^^]^. Consistent with our data, some behavioral and biochemical findings have demonstrated that the activity of P2X4 receptors expressed in dorsal horn microglia is necessary for the induction of mechanical allodynia^[^^38 ▶^^,^^39 ▶^^]^. A number of investigations have revealed that BDNF neurotrophin has a critical role in neuropathic pain and its expression boosts in the spinal cord dorsal horn and DRG^[^^40 ▶^^,^^41 ▶^^]^. The activation of the P2X4 receptor in microglia is essential for the expression and release of BDNF after peripheral nerve injury, which in turn leads to the increased pain transfer in neurons^[^^19 ▶^^]^. It has also been suggested that BDNF indirectly facilitates the release of GABA from the spinal cord interneurons^[^^22 ▶^^]^, and *BDNF* expression increases in the spinal cord (dorsal horn) 24 hours after SNL, and this elevation continues for several days^[40]^. In the present study, while after CCI, the expression of the *BDNF* gene enhanced significantly, paroxetine reduced the expression of this neurotrophin both before and after the nerve injury. Our previous surveys demonstrated that the prophylactic injection of paroxetine could diminish neuropathic pain^[^^42 ▶^^]^. Considering the key role of BDNF in neuropathic pain, this change in pain behavior is probably due to a shift in *BDNF *expression, which is reduced by paroxetine when administered before and after nerve damage. 

KCC2 is a transporter for potassium and chloride ions and contributes to the regulation of anion gradients on both sides of the membrane. This transporter plays a very important role in regulating GABAA receptor activity^[^^43 ▶^^]^. The post-synaptic activity of GABAA receptors in the adult nervous system leads to the opening of the chloride channel, neuronal hyperpolarization and as a result, its inhibitory activity^[^^44 ▶^^,^^45 ▶^^]^. The role of KCC2 has been proven in chlorine ion homeostasis in the spinal interneurons. KCC2 actively pumps the ion chloride to the outside of the neuronal cell to support conditions for the inhibitory activity of GABA receptors^[^^46 ▶^^,^^47 ▶^^]^. Moreover, the expression of KCC2 can contribute to the neuropathic pain induced by nerve injury^[^^6 ▶^^,^^48 ▶^^,^^49 ▶^^]^. In this study, paroxetine increased the expression of *KCC2* as compared to the control group when used before or after nerve injury. Further researches are needed to find out whether paroxetine directly affects the expression of this protein, or secondarily, by blocking the purinergic receptor that causes this change. The GABAA receptors existing at the end of the primary afferent neurons are responsible for their synaptic inhibition^[^^50 ▶^^,^^51 ▶^^]^. It has been proven that GABAA*/*_2_ expression decreases significantly in the DRG after the nerve injury^[^^25 ▶^^]^. However, after the nerve injury, the expression of *BDNF* rises in DRG neurons, but the γ_2_subunit of GABAA receptors decreases concurrently^[^^52 ▶^^]^. As mentioned before, KCC2, as a key protein in regulating the equilibrium potential of anions, is crucial for the GABAA inhibitory effect^[^^53 ▶^^]^. Reduced expression of the KCC2 protein in dorsal horn of the spinal cord neurons leads to the elimination of the inhibitory function of GABAA receptor in case of chronic neuropathy^[^^23 ▶^^,^^54 ▶^^]^. However, considering what mentioned before, the inhibitory effect of GABAA receptor depends on two important factors: first, the expression of the receptor itself and its subunits, and the second, the post-synaptic activity of the membrane protein of KCC2. The findings of this study showed that only pre-injury injection of paroxetine resulted in the increased protein level of GABAA*/*_2_ receptors. 

In conclusion, it seems that paroxetine with change in the expression of some important proteins involved in neuropathic pain (BDND, KCC2, and GABAA) attenuates neuropathic pain. This effect was observed either when paroxetine was administered before nerve injury or when it was injected after damage to the nerve.
